# A Transgenic Mouse Model of Pacak–Zhuang Syndrome with An *Epas1* Gain-of-Function Mutation

**DOI:** 10.3390/cancers11050667

**Published:** 2019-05-14

**Authors:** Herui Wang, Jing Cui, Chunzhang Yang, Jared S. Rosenblum, Qi Zhang, Qi Song, Ying Pang, Francia Fang, Mitchell Sun, Pauline Dmitriev, Mark R. Gilbert, Graeme Eisenhofer, Karel Pacak, Zhengping Zhuang

**Affiliations:** 1 Neuro-Oncology Branch, Center for Cancer Research, National Cancer Institute, Bethesda, MD 20892, USA; herui.wang@nih.gov (H.W.); jing.cui@nih.gov (J.C.); chungzhang.yang@nih.gov (C.Y.); jared.rosenblum@nih.gov (J.S.R.); zhangqi86@gmail.com (Q.Z.); qisong725@gmail.com (Q.S.); pauline.dmitriev@nih.gov (P.D.); mark.gilbert@nih.gov (M.R.G.); 2 Eunice Kennedy Shriver National Institute of Child Health and Human Development, National Institutes of Health, Bethesda, MD 20892, USA; ying.pang@nih.gov; 3 Surgical Neurology Branch, National Institute of Neurological Diseases and Stroke, National Institutes of Health, Bethesda, MD 20892, USA; franciafang@gmail.com (F.F.); mitchsun12@gmail.com (M.S.); 4 Institute of Clinical Chemistry and Laboratory Medicine and Department of Medicine III, University Hospital Carl Gustav Carus, Technische Universität Dresden, 01307 Dresden, Germany; Graeme.Eisenhofer@uniklinikum-dresden.de

**Keywords:** paraganglioma, somatostatinoma, polycythemia, *EPAS1*, transgenic mice, erythropoietin

## Abstract

We previously identified a novel syndrome in patients characterized by paraganglioma, somatostatinoma, and polycythemia. In these patients, polycythemia occurs long before any tumor develops, and tumor removal only partially corrects polycythemia, with recurrence occurring shortly after surgery. Genetic mosaicism of gain-of-function mutations of the *EPAS1* gene (encoding HIF2α) located in the oxygen degradation domain (ODD), typically p.530–532, was shown as the etiology of this syndrome. The aim of the present investigation was to demonstrate that these mutations are necessary and sufficient for the development of the symptoms. We developed transgenic mice with a gain-of-function *Epas1^A529V^* mutation (corresponding to human *EPAS1^A530V^*), which demonstrated elevated levels of erythropoietin and polycythemia, a decreased urinary metanephrine-to-normetanephrine ratio, and increased expression of somatostatin in the ampullary region of duodenum. Further, inhibition of HIF2α with its specific inhibitor PT2385 significantly reduced erythropoietin levels in the mutant mice. However, polycythemia persisted after PT2385 treatment, suggesting an alternative erythropoietin-independent mechanism of polycythemia. These findings demonstrate the vital roles of *EPAS1* mutations in the syndrome development and the great potential of the *Epas1^A529V^* animal model for further pathogenesis and therapeutics studies.

## 1. Introduction

We previously identified a novel syndrome (also known as Pacak–Zhuang Syndrome) characterized by the clinical constellation of paraganglioma, somatostatinoma, and polycythemia. Several features in this syndrome are unique and clustered [1,2]. First, the lack of family history of similar symptoms or pathologies suggests a non-hereditary pattern. Second, the syndrome demonstrates female sex predominance. Third, patients demonstrate early onset polycythemia, presenting at birth. Fourth, all patients develop several rare tumors, including paraganglioma (PGL) and somatostatinoma, which we suspected would be unlikely without a common underlying genetic pathogenesis [1,2].

We found that the patients share common postzygotic mutations, including p.A530T/V, P531S, Y532C, L529P, T519M, and P544S, in the oxygen degradation domain (ODD) of *EPAS1*, encoding hypoxia-inducible factor 2α (HIF2α) [1]. These mutations were found to disturb the hydroxylation of ODD of the HIF2α protein by prolyl hydroxylase 2 (PHD2), which impairs its binding with von Hippel–Lindau protein and subsequently increases HIF2α protein stability [1]. This leads to increased transcription of the genes downstream of the HIF2α/HIF1β dimer in the tumors, such as *EPO*, *VEGFA*, *SLC2A1*, and *VPS11* [2], which causes pseudohypoxia signaling and influences the developmental physiology and disease pathology of the syndrome.

PGLs are rare catecholamine-producing tumors that are derived from chromaffin cells of extra-adrenal paraganglia; somatostatinoma is also of neural crest origin. PGLs are classified into two expression clusters: (1) Cluster 1 with high *EPAS1* expression and immature phenotypic features, (2) Cluster 2 with low *EPAS1* expression and mature phenotypic features [3]. Patients with Pacak–Zhuang syndrome consistently fall into Cluster 1 and are found to have high levels of normetanephrine (NMN) and norepinephrine (NE) [1].

Polycythemia is an abnormal elevation of the hematocrit caused by either increased production or decreased destruction of red blood cells (RBCs). Secondary polycythemia occurs as a consequence of elevated circulating erythropoietin (EPO), while primary polycythemia is due to intrinsic factors (e.g., somatic *JAK2^V617F^* mutation and hereditary dominant *EPOR* mutations) of erythroid progenitors in the bone marrow and is EPO-independent [4]. Mixed polycythemia, such as Chuvash polycythemia caused by *VHL^R200W^* mutation, has features of both primary and secondary polycythemias characterized by elevated EPO and erythroid progenitors hypersensitive to EPO [5]. Elevated plasma EPO confirmed secondary polycythemia in the syndrome patients, but it is still unclear whether primary polycythemia exists.

Hypoxia signaling pathways have been established as critical to disease pathogenesis as well as normal development [6,7,8,9]. *EPAS1* mutations were previously only found to cause familial polycythemia and pulmonary arterial hypertension [10,11,12]. This new syndrome of paraganglioma, somatostatinoma, and polycythemia provides a unique opportunity to study the impact of hypoxia signaling, specifically gain-of-function of HIF2α, on tumorigenesis.

In this study, we aimed to develop a transgenic mouse model to achieve the following aims: (1) to confirm *EPAS1* mutations are causative gene mutations for the syndrome and (2) to use this model for further pathogenesis and therapeutic studies of the syndrome.

## 2. Results

### 2.1. Establishment of A Somatic Epas1^A529V^ Animal Model

**Figure 1:**
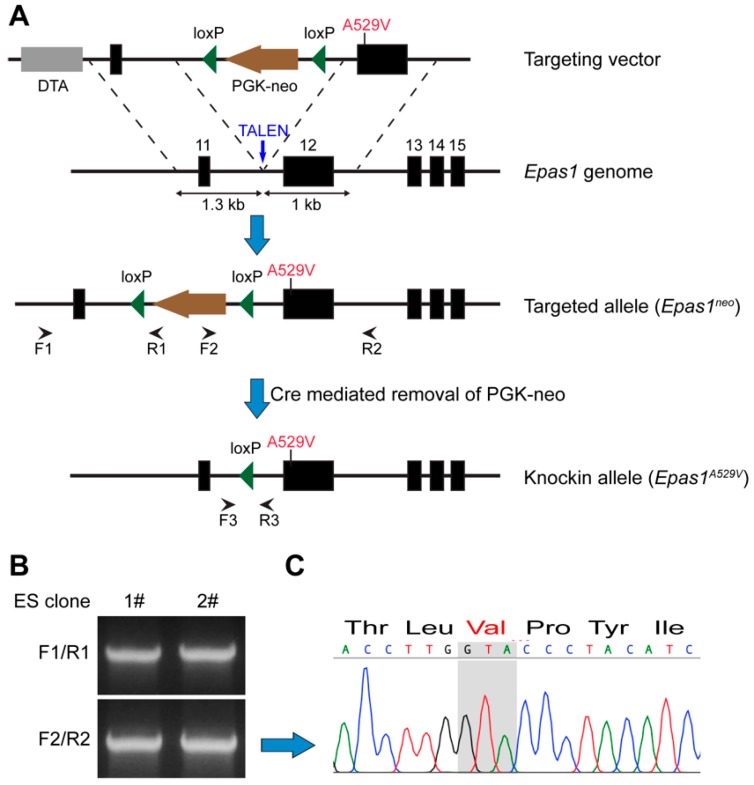
Establishment of the *Epas1^A529V^* animal model. (**A**) Schematic strategy of the mutant mice generation. (**B**) Positive embryonic stem (ES) colonies were confirmed by PCR at both 5′ (F1/R1) and 3′ (F2/R2) ends. (**C**) Sanger sequencing result of the F2/R2 PCR band. The mutant codon is labeled in red.

The syndrome patients were found to carry somatic *EPAS1* mutations in the ODD without other germline mutations [2]. We thus generated a transgenic mouse model with a somatic heterozygous *Epas1^A529V^* mutation (corresponding to human *EPAS1^A530V^*). Transcription activator-like effector nucleases (TALEN) were utilized to facilitate homologous recombination in the embryonic stem (ES) cells (Figure 1A). The targeting vector contained 1.3 kb 5′ and 1 kb 3′ homology arms, neomycin selection, and diphtheria toxin A negative selection cassettes. *Epas1^A529V^* point mutation is located in the 3′ homology arm. G418-resistant ES cell colonies were picked up after co-electroporation of TALEN expression vectors and *Epas1* A529V targeting vector into B6:129-mixed-background ES cells. Positive recombinant ES colonies were confirmed by PCR at both 5′ and 3′ ends (Figure 1B). Sanger sequencing also confirmed the presence of the A529V mutation (GCA>GTA) in the positive ES colonies before injection into the blastocysts (Figure 1C). Chimera and subsequent germline-transmitted mice (*Epas1^neo/+^*) were derived. The neomycin cassette upstream of the A529V point mutation in exon 12 blocked the transcription of the mutant allele, and no obvious defects were observed in *Epas1^neo/+^* mice.

**Figure 2:**
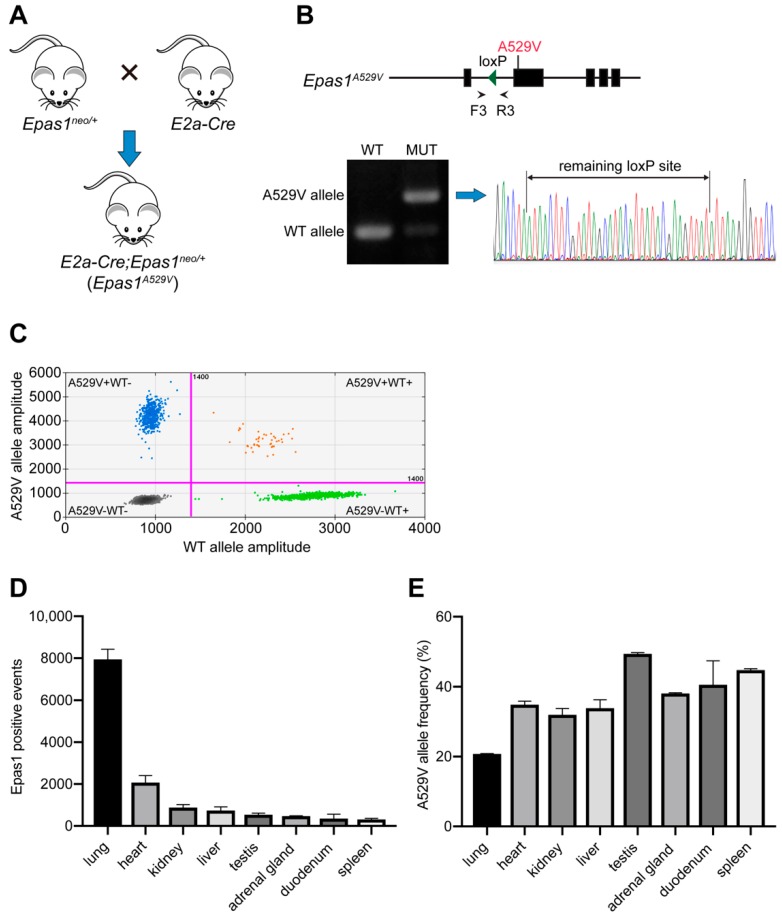
Successful expression of *Epas1^A529V^* mutant allele in various tissues. (**A**) Mouse breeding strategy to generate the somatic mutant mice. (**B**) Genotyping PCR (F3/R3) and Sanger sequencing confirmed the successful deletion of the neomycin cassette by *E2a-Cre* in one-month-old *Epas1^A529V^* mutant mice. (**C**) Representative image of *Epas1^A529V^* droplet digital PCR (ddPCR). Green dots, droplets with PCR amplification of *Epas1* wild-type (WT) allele. Blue dots, droplets with PCR amplification of *Epas1* A529V mutant (MUT) allele. Orange dots, droplets with PCR amplification of both alleles. (**D**) Total *Epas1*-positive events of *Epas1* ddPCR from 100 ng cDNA of each tissue in two–three-month-old male mutant mice. *n* = 3. (**E**) *Epas1^A529V^* allele frequency in the cDNA derived from each tissue.

To activate the expression of the A529V mutant allele, we mated *Epas1^neo/+^* mice with *E2a-Cre* transgenic mice in C57BL/6 background and generated somatic heterozygous *Epas1^A529V^* mutant mice (*E2a-Cre*; *Epas1^neo/+^*, in brief, *Epas1^A529V^*) (Figure 2A). Genotyping PCR and Sanger sequencing confirmed the successful deletion of the neomycin cassette in tail DNA of *Epas1^A529V^* mutant mice (Figure 2B). To confirm the expression of the *Epas1^A529V^* mutant allele, we extracted RNA from multiple tissues of the *Epas1^A529V^* mutant mice, including heart, lung, liver, kidney, duodenum, adrenal gland, spleen, and testis, and performed reverse transcription. Droplet digital PCR (ddPCR) with complementary DNA (cDNA) of each tissue confirmed high expression of *Epas1* in lung and heart (Figure 2C,D), consistent with a previous report [13]. The percentage of *Epas1^A529V^* mutant allele in cDNA varied from 20.8% to 49.4% in different tissues (Figure 2E). These results confirmed Cre-mediated high expression of *Epas1^A529V^* mutant allele in a wide range of tissues.

### 2.2. Polycythemia and Elevated EPO in Epas1^A529V^ Mutant Mice

**Figure 3:**
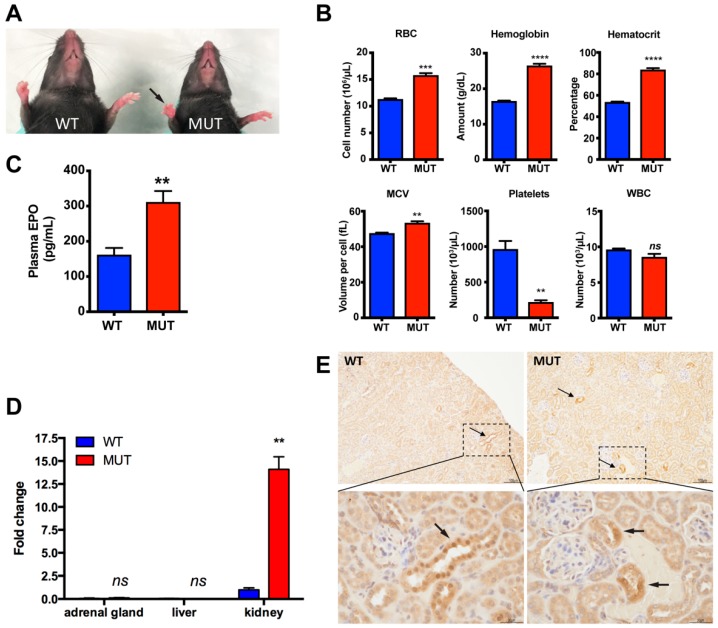
Polycythemia and elevated erythropoietin (EPO) in *Epas1^A529V^* mutant mice. (**A**) Red palm (arrow) in three-month-old mutant mice. (**B**) Complete blood count (CBC) test confirmed polycythemia in two-month-old mutant mice. MUT, *Epas1^A529V^* mutant mice. WT, littermate control mice. *n*(WT) = 4, *n*(MUT) = 3; ns, *p* > 0.05; ** *p* < 0.01; *** *p* < 0.001; **** *p* < 0.0001. (**C**) Elevated plasma EPO in *Epas1^A529V^* mutant mice. ** *p* < 0.01. (**D**) *Epo* expression in different tissues of four-month-old mice; *n* = 3 for each group. (**E**) EPO immunohistochemistry (IHC) staining of control and mutant kidney. Arrows indicate EPO-positive cells. RBC: red blood cells, MCV: mean corpuscular volume, WBC: white blood cells. Scale bars: top, 100 µm, bottom, 30 µm.

Red palms in *Epas1^A529V^* mutant mice suggested an underlying polycythemia (Figure 3A). A complete blood count (CBC) test confirmed polycythemia by respective 39.9%, 60.7%, and 56.5% elevations in erythrocyte count, hemoglobin, and hematocrit of somatic *Epas1^A529V^* mutant mice compared to littermate controls (Figure 3B). Minorly increased mean corpuscular volume (MCV) and significantly reduced platelets in the mutant mice were noted, and no change was observed for white blood cells (Figure 3B).

We measured plasma EPO concentrations and observed significantly increased EPO levels in *Epas1^A529V^* mice (Figure 3C). The EPO concentrations in mutant mice were about twice those in littermate control mice. Elevated plasma EPO level in mutant mice is expected because the *Epo* gene is a direct target of the HIF2α/HIF1β dimer [14,15]. We also performed real-time RT-PCR to compare *Epo* mRNA levels in different tissues and found that *Epo* expression was much higher in kidney than in other tissues of both control and mutant mice (Figure 3D). *Epo* expression level was dramatically enhanced in mutant kidney by about thirteen-fold compared to control kidney (Figure 3D). EPO immunohistochemistry (IHC) staining also confirmed increased EPO expression in the mutant kidney (Figure 3E). These results suggest that the *Epas1^A529V^* mutation increased EPO expression in the kidney, leading to polycythemia.

### 2.3. Biochemistry Characteristics of Epas1^A529V^ Mutant Mice

**Figure 4:**
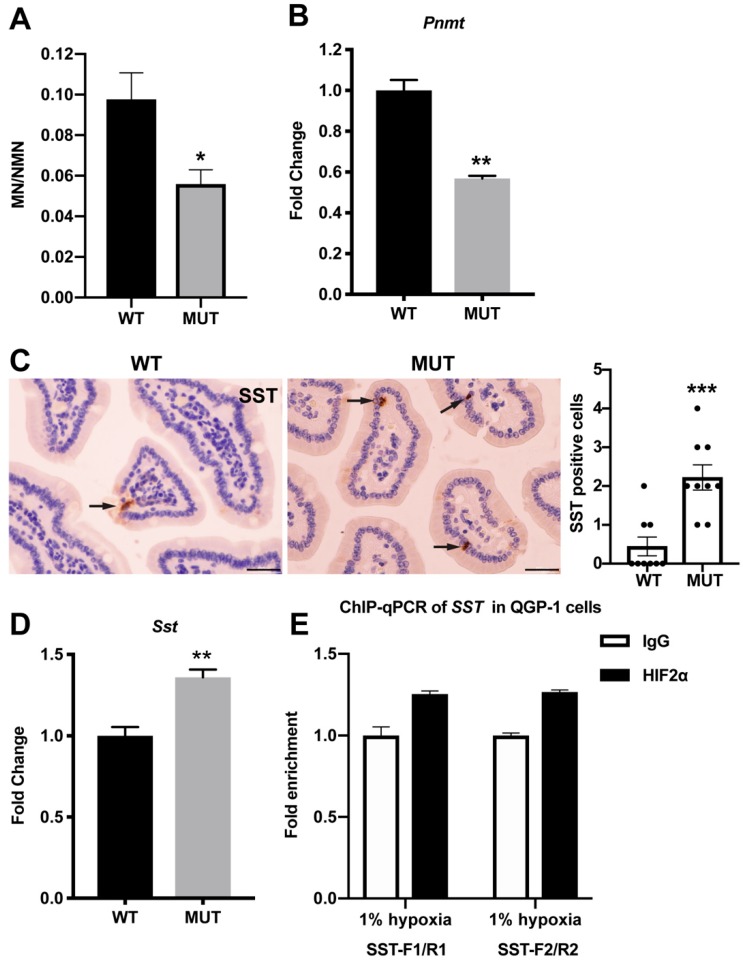
*Epas1^A529V^* mutant mice recaptured the biochemistry characteristics of the syndrome. (**A**) Decreased metanephrine (MN)/normetanephrine (NMN) ratio in three–five-month-old mutant mice. *n*(WT) = 6, *n*(MUT) = 7. * *p* < 0.05. (**B**) Decreased *Pnmt* mRNA in mutant adrenal gland. ** *p* < 0.01. (**C**) SST IHC staining of duodenum of control and mutant mice. Arrows indicate SST-positive cells. SST-positive cells were counted in nine random fields of view (400×) and summarized in the right column. *** *p* = 0.0005. Scale bars, 30 µm. (**D**) Increased *Sst* mRNA in mutant duodenum; *n* = 3 for each group. (**E**) ChIP qPCR with an HIF2α antibody or Rabbit IgG in QGP-1 cells.

There is no suitable animal model with spontaneous development of paraganglioma and somatostatinoma [16,17]. Thus, we sought to develop paraganglioma and somatostatinoma in *Epas1^A529V^* mutant mice. Although no pheochromocytomas or paragangliomas were found in up to one-year-old mutant mice, lower ratios of urinary metanephrine (MN) to NMN were observed in *Epas1^A529V^* mutant mice (Figure 4A). Expression of phenylethanolamine N-methyltransferase (PNMT), which converts norepinephrine (precursor of NMN) to epinephrine (precursor of MN) was similarly down-regulated in the adrenal glands of mutant mice (Figure 4B). These observations are consistent with previous findings that HIF2α negatively regulates PNMT expression and is thereby responsible for the immature noradrenergic features of chromaffin cell tumors with high *EPAS1* expression [3,18].

In patients with this syndrome, somatostatinoma always appears in the ampullary region of the duodenum [2]. Somatostatin IHC staining confirmed more positive cells in the duodenum of mutant mice than in littermate control mice (Figure 4C). Although gross somatostatinoma was not found in our mice, enhanced expression of *Sst*, encoding somatostatin, was found in the duodenum tissue of *Epas1^A529V^* mutant mice (Figure 4D). To check whether HIF2α binds to the promoter region of *SST*, we performed ChIP-qPCR with an HIF2α antibody in the human pancreatic islet cell carcinoma (somatostatinoma) cell line QGP-1. Both *SST* primer pairs in the *SST* promoter region confirmed that HIF2α can bind to the hypoxia response element (HRE) in the *SST* promoter (Figure 4E). These results suggest that *SST* may be a potential target of the HIF2α/HIF1β dimer.

### 2.4. Inhibition of HIF2α Reduced EPO but Not Polycythemia in Epas1^A529V^ Mutant Mice

**Figure 5:**
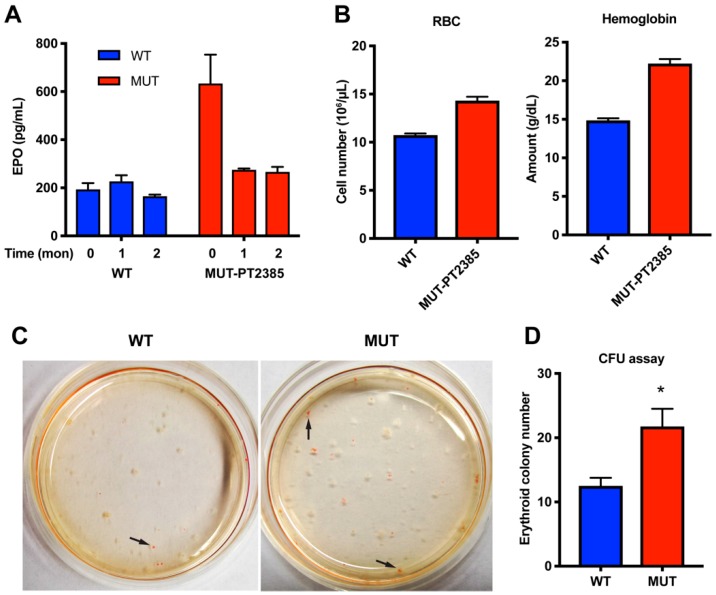
HIF2α inhibition in the mutant mice. (**A**,**B**) PT2385 reduced EPO (**A**) but not polycythemia (**B**) in three–four-month-old *Epas1^A529V^* mutant mice; *n* = 3 for each group. (**C**) Representative image of the colony-forming unit (CFU) assay. Arrows indicate the erythroid colonies. (**D**) Summary of the erythroid colonies from bone marrow CFU assay; *n* = 3 for each group. * *p* < 0.05.

Treatment of the mutant mice for one month with a specific antagonist of HIF2α, PT2385, demonstrated effective reduction of EPO levels in the mutant mice (Figure 5A). However, this antagonism of HIF2α did not resolve polycythemia even after treatment for two months (Figure 5B). We thus investigated an alternative mechanism causing polycythemia not responsive to EPO level reduction with transient treatment. The colony-forming unit (CFU) assay of bone marrow hematopoietic progenitors revealed increased erythroid colony number in mutant mice (Figure 5C,D), indicating that the gain-of-function mutant HIF2α increased the erythroid differentiation of the progenitor cells and further supporting an EPO-independent component of polycythemia in this syndrome [19].

## 3. Discussion

In this study, we successfully generated a transgenic animal model mimicking postzygotic *EPAS1* mutations. These mice share polycythemia and biochemistry features of the Pacak–Zhuang syndrome. Inhibition of HIF2α with its specific inhibitor, PT2385, significantly reduced EPO. Increased erythroid colony number in the CFU assay in mutant mice indicates that somatic *Epas1^A529V^* mutation in erythrocyte progenitor cells of the bone marrow may also contribute to primary polycythemia in the syndrome. Thus, the *Epas1^A529V^* mutant animal model has great potential for further pathogenesis and therapeutic studies of the syndrome.

HIF2α plays an essential, tightly regulated role in development [20]. Stabilization of HIF2α due to gain-of-function mutations may impact organ systems of neural crest origin, according to the time point during early development. Somatostatin and adrenal medullary cells are of neural crest origin; early mutation of *Epas1* may impact the migration path of these cells and lead to tumors characterized by clusters of immature cells. The *Epas1^A529V^* mouse model developed in the present study supports this mutation as the etiology of the Pacak–Zhuang syndrome. We have demonstrated that the mutation is sufficient for the development of polycythemia, increased EPO secretion, somatostatinoma-related manifestations, and immature chromaffin cell features consistent with the origins of the noradrenergic paragangliomas and pheochromocytomas characteristic of the affected patients. The developmental role of *Epas1* and its regulation by signaling cascades of neurulation provide a probable mechanistic rationale for the long duration of tumor development.

Notably, the overproduction of erythrocytes and decreased platelets in somatic heterozygous *Epas1^A529V^* mice were more significant than what was observed in *Epas1^G536W/G536W^* mice [12]. This indicates that our model also mirrors human polycythemias with gain-of-function *EPAS1* mutations that are inherited in a dominant fashion (heterozygote). The proximity of A529 to P530, the hydroxylation site of PHD2, compared to G536, appears to result in a more severe phenotype, suggesting that severity is related to the degree of impact on the association of PHD2 with HIF2α.

PGL patients of Pacak–Zhuang syndrome consistently fall into Cluster 1 with very high plasma levels of NMN and NE and relatively normal levels of MN and epinephrine (EPI) [1,2]. These results suggest that stabilized HIF2α protein caused by gain-of-function mutations in the ODD domain is sufficient to block the differentiation of chromaffin progenitor cells and maintain their immature phenotype. In patients with this syndrome, the somatostatinoma always appears in the ampullary region of the duodenum and not in the pancreas. Although no discrete paraganglioma or somatostatinoma tumors were found in the mutant mice, we confirmed *Sst* increase in the duodenum but not in the pancreas, which supports cluster formation of immature cells with up-regulated *Sst* in the duodenum of the patients as the mechanism for this neuroendocrine tumor.

Unlike polycythemia, which is present from birth in both patients with the syndrome and mutant mice, Pacak–Zhuang syndrome patients develop paragangliomas and somatostatinoma in their early thirties. We believe that the development of discrete tumors may be more complicated and likely depends on multiple factors including acquiring additional driver gene mutations (e.g., copy number alterations of 1p *SDHB* and 3p *VHL*) and environment changes such as hypoxic stress [21]. Additional tests are necessary in the future to determine what factors are required to trigger tumor development in the *Epas1* gain-of-function mouse model.

## 4. Materials and Methods

### 4.1. Mouse Model and Genotyping

Briefly, two adjacent homologous arms were inserted into the Kpn1/Sal1 and Mlu1/Not1 sites of PGKneolox2DTA.2 (a gift from Philippe Soriano (Icahn School of Medicine at Mount Sinai, New York, NY, USA), Addgene plasmid #13449), respectively. The 5′ homologous recombination (HR) arm is a 1.3 kb PCR product (Kpn1-Forward: CGGGGTACCAGTAGATACTCAGGGACACCCAT, Sal1-Reverse: ACGCGTCGACAGTAGATACTCAGGGACACCCAT). The 3′ HR arm is adjacent to the 5′ HR arm sequence and is a 1 kb PCR product including exon 12 (Mlu1-Forward: CGACGCGTGGTGAGTGAGAACAGCAGTCCC, Not1-Reverse: AAGGAAAAAAGCGGCCGCATAAGCAGGTGTGTACATGTA). *Epas1* A529V point mutation was then introduced in the 3′ arm of the HR vector using QuikChange Lightning Site-Directed Mutagenesis Kit (Agilent, Santa Clara, CA, USA). TALEN vectors were assembled following ZiFiT instruction (http://zifit.partners.org/ZiFiT/Disclaimer.aspx). The HR vector and two TALEN vectors were electroporated into mouse ES cells at a ratio of 2:1:1. ES colonies were picked up after G418 selection (200 ug/mL) for 10 days and further identified by PCR and Sanger sequencing. Identification primers for the 5′ end: F1: CTACACCCAGTGCTTCAAG, R1: TGAGGCGGAAAGAACCA. Identification primers for the 3′ end: F2: CGAAGGAGCAAAGCTGCTA, R2: AAAGTGCCAGCTGCCTACACATAC. The ES colony with correct recombination was then micro-injected into mouse blastocysts to generate chimeric mice.

*E2a-Cre* transgenic mice with B6 background were kindly provided by Alex Grinberg of Eunice Kennedy Shriver National Institute of Child Health and Human Development. Progeny carrying the mutant genotype (*E2a-Cre*; *EPAS1^neo/+^*, in brief, *Epas1^A529V^*) was acquired. Littermate control mice were used for all experiments.

### 4.2. Complete Blood Count (CBC)

Mouse facial vein blood was collected in K2EDTA tubes (BD Microtainer, Franklin Lakes, NJ, USA). A total of 90 μL whole blood was diluted with 180 μL normal saline before sending to the Department of Laboratory Medicine at Clinical Center of NIH for complete blood count.

### 4.3. Enzyme-Linked Immunosorbent Assay (ELISA)

EPO in the mouse plasma was determined using an ELISA kit according to the manufacturer’s instructions (R&D systems, Minneapolis, MN, USA). Briefly, facial vein blood was collected in heparin tubes, and plasma was collected by centrifuging at 10,000 g for 10 min at 4 °C and was frozen immediately at −80 °C. Plasma was thawed on ice when used and was diluted to 1:2–1:4 (depending on the volume of the plasma) for experiments. The samples were assayed in duplicates.

### 4.4. Quantitative Real-Time Polymerase Chain Reaction (qRT-PCR)

RNA of the indicated tissues was extracted with a Purelink RNA Mini Kit (Thermo Fisher Scientific, Waltham, MA, USA). Totally, 500 ng RNA was reverse-transcribed with iScript CDNA Synthesis kit (Bio-Rad, Hercules, CA, USA). The reactions were prepared with SsoAdvanced universal SYBR Green supermix (Bio-Rad) and were run on a CFX384 real-time system (Bio-Rad). Primers used for qRT-PCR included Epo (forward: ATGAAGACTTGCAGCGTGGA, reverse: TTCTGCACAACCCATCGTGA), Pnmt (forward: AAGTCAACCGTCAGGAGCTG, reverse: TCGAAGCTGGCGTTCTTTCT), Sst (forward: AGCTGGCTGCAAGAACTTCT, reverse: AGGGTCAAGTTGAGCATCGG), and Actb (forward: GACCTCTATGCCAACACAGT, reverse: AGTACTTGCGCTCAGGAGGA).

### 4.5. Immunohistochemistry (IHC) Staining

IHC staining was performed as previously described [2]. The primary antibodies used in this study were anti-EPO (Santa Cruz Biotechnology, Dallas, TX, USA; sc-7956, 1:100) and anti-SST (Abcam, Cambridge, MA, USA; ab30788, 1:100).

### 4.6. Droplet Digital PCR (ddPCR)

ddPCR was performed with the BioRad QX200 ddPCR system in the Genomics Core Facility of the NCI Center for Cancer Research (CCR), according to the manufacturer’s instructions. ddPCR mutation assay of *Epas1^A529V^* was designed on the basis of the mouse *Epas1* coding sequence with the Bio-Rad website tool. The unique assay ID is dMDS358400990. The probe for the wild-type allele was labelled with Hexachloro (HEX) fluorescence, and the probe for the A529V mutant allele was labelled with Fluorescein (FAM) fluorescence. In total, 100 ng cDNA of each tissue was used for ddPCR reaction, and the results were analyzed with the QuantaSoft software (Bio-Rad). *Epas1* gene expression level in different tissues was compared by combining positive events of both wild-type and mutant alleles. *Epas1^A529V^* allele frequency was compared by the fractional abundance.

### 4.7. PT2385 Treatment

Three–four months old mice were selected for PT2385 treatment. Before treatment, plasma from facial vein blood of each mouse was collected as the basal level. PT2385 was dissolved in DMSO at 10 mM as stock solution. During treatment, the PT2385 stock solution was diluted with normal saline and intraperitoneally administrated to the mice every other day at a concentration of 400 μg/kg body weight. Plasma of facial vein blood was collected every month for the determination of EPO levels.

### 4.8. Determinations of Urinary Catecholamines and Metanephrines

Mouse urine was collected for measurements of catecholamines and metanephrines by liquid chromatography with mass spectrometry, as described previously [22].

### 4.9. ChIP-qPCR

ChIP assays were performed using SimpleCHIP Enzymatic Choromatin IP Kit (Magnetic beads) following the manufacturer’s instructions (Cell Signaling Technology, Danvers, MA, USA; catalog 9003). Briefly, cross-linked protein-DNA complexes were precipitated by incubation with rabbit anti-HIF2α (Abcam; ab199) or rabbit IgG (negative control) overnight and then with magnetic beads for 2 hours. The purified DNA fragments including HIF-binding element (HRE) were quantitatively analyzed by real-time PCR with primers against the *SST* promoter (hSST-HRE-F1/B1 and F2/B1) following the standard-curve method. The standard curves were created by serial dilution of 2% input chromatin DNA. The values of chromatin DNA precipitated by HIF2α antibody were normalized to those precipitated by normal rabbit IgG, which was arbitrarily defined as 1. The primer sequences are: hSST-HRE-F1: ATCGTGGGGCATGTGGAATT; hSST-HRE-F2: AATCGTGGGGCATGTGGAAT; hSST-HRE-B1: TGTGTGCTCTCAACCGTCTC.

### 4.10. Colony-Forming Unit (CFU) Assay

The CFU assay was performed according to the manufacturer’s instruction (R&D Systems, Minneapolis, MN, USA; HSC007). Briefly, 30,000 bone marrow cells of one-year-old *Epas1^A529V^* or control mice were plated in 35 mm cell culture dishes with methylcellulose-based media. Reddish colonies in each dish were counted after one week. Duplicate dishes were used for each mouse. Statistics was performed by the unpaired Student’s *t*-tests.

### 4.11. Statistics

Data are shown by mean with SEM. The *p* values were calculated using Student’s *t*-test; *p* values of less than 0.05 were considered statistically significant.

### 4.12. Study Approval

All in vivo experiments were performed under the animal protocol (NICHD 18-028) that was reviewed and approved by the Animal Care and Use Committee of NICHD.

## 5. Conclusions

Our somatic heterozygous *Epas1^A529V^* mutant mouse model is the first animal model of the syndrome of paraganglioma, somatostatinoma, and polycythemia. These mice share polycythemia and biochemistry features of the syndrome, demonstrating gain-of-function mutations of *EPAS1* in the ODD domain as the causative gene mutation of the syndrome development. This mutant animal model has great potential for further pathogenesis and therapeutics studies of the syndrome.
